# Derivatives of *Dictyostelium* Differentiation-Inducing Factor-3 Promote Autophagic Flux, Mitochondrial Dysfunction and Iron-Associated Oxidative Stress

**DOI:** 10.3390/ijms27209380

**Published:** 2026-10-21

**Authors:** Yue Cai, Ryu Yamada, Yuki Marugame, Satoshi Yano, Fumihiko Okumura, Haruhisa Kikuchi, Yuzuru Kubohara, Taichi Hara

**Affiliations:** 1 Laboratory of Food and Life Science, Institute of Human Sciences, Waseda University, Saitama 359-1192, Japan; caiyue1999@akane.waseda.jp (Y.C.);; 2 Department of Food and Health Sciences, International College of Arts and Sciences, Fukuoka Women’s University, Fukuoka 813-8529, Japan; 3 Division of Natural Medicines, Faculty of Pharmacy, Keio University, Tokyo 105-8512, Japan; halkiku@keio.jp; 4 Faculty of Health and Sports Science, Juntendo University, Chiba 270-1695, Japan

**Keywords:** *Dictyostelium discoideum*, differentiation-inducing factor, autophagy, mTORC1, iron-associated oxidative stress, reactive oxygen species

## Abstract

Novel anticancer agents are needed to overcome therapeutic resistance, and autophagy represents a promising target. The chlorinated alkylphenones *Dictyostelium* differentiation-inducing factors 1 and 3 (DIF-1 and DIF-3) and their derivatives exert anti-proliferative and anti-metastatic effects; however, their molecular mechanisms remain incompletely understood. Using the GFP-LC3-RFP-LC3ΔG autophagic flux reporter, we identified Bu-DIF-3 and CP-DIF-3 as autophagy-promoting derivatives and characterized their actions principally in HeLa cells, with selected autophagy and AMP-activated protein kinase (AMPK) measurements in HAP1 cells. Both suppressed mTORC1 signaling, activated AMPK, and reduced mitochondrial membrane potential (MMP). Bu-DIF-3 additionally induced reactive oxygen species and redox stress response genes, including *SLC7A11* and *CHAC1*. *SLC7A11* induction may reflect a compensatory antioxidant response. Unlike CP-DIF-3, Bu-DIF-3 increased the FerroOrange-detectable labile Fe^2+^ signal, and deferoxamine partially restored the reduced MTT signal, supporting possible iron involvement in reduced metabolic activity. Thus, modification of the DIF-3 methoxy group modulates autophagy-promoting activity and associated stress responses. Bu-DIF-3 was associated with oxidative stress and altered labile Fe^2+^ homeostasis; whether a ferroptosis-related mechanism contributes remains unresolved. These findings provide a cellular basis for further evaluation of Bu-DIF-3 and related DIF derivatives.

## 1. Introduction

Natural products remain a rich source of anticancer lead compounds with novel mechanisms of action [1]. Autophagy is an evolutionarily conserved intracellular degradation system essential for cellular homeostasis, and its dysregulation is implicated in various diseases, including neurodegeneration and cancer [2,3]. This process involves the formation of double-membraned autophagosomes that sequester damaged organelles, misfolded proteins, and invading pathogens, followed by their degradation after fusion with lysosomes [4]. During tumor initiation and progression, autophagy plays a “double-edged sword” role: it can promote tumor cell survival by facilitating stress adaptation and metabolic reprogramming, but it may also contribute to tumor suppression or cell death induction under specific conditions [5]. Accordingly, precise modulation of autophagic activity has emerged as a highly promising strategy for cancer therapy.

Differentiation-inducing factors 1 and 3 (DIF-1 and DIF-3) are chlorinated alkylphenones that were originally identified as inducers of stalk cell differentiation in the cellular slime mold *Dictyostelium discoideum* [6,7]. This differentiation process is regarded as a form of programmed autophagic cell death [8]. DIFs and their derivatives exert diverse biological activities in mammalian cells [9,10,11,12,13,14,15], including potent anti-tumor effects, making them attractive lead compounds for anticancer drug development [9,12,13,14,15,16,17,18,19,20,21,22]. Several DIF compounds exert marked anti-proliferative, anti-metastatic, and/or cytotoxic effects against various human cancer cells, including breast cancer, leukemia, and melanoma cells [16,17,23,24,25]. Accumulating evidence suggests that the anti-tumor effects of DIF compounds involve multiple molecular targets and signaling pathways. For example, DIFs increase intracellular Ca^2+^ levels in several tumor cell types [26,27,28]. In addition, DIFs directly inhibit bovine calmodulin-dependent cyclic nucleotide phosphodiesterase in vitro [29]. In MCF-7 breast cancer cells, DIF-1 suppresses cell proliferation by inhibiting the protein synthesis of ribosomal protein S6 kinase (S6K)-mediated signal transducer and activator of transcription 3 [16]. In more aggressive triple-negative breast cancer models, DIF-1 suppresses tumor growth and metastasis by activating AMP-activated protein kinase (AMPK) and subsequently inhibiting the mTORC1–S6K signaling pathway [17]. In addition, DIF compounds may exert various biological activities by inhibiting mitochondrial function. In several tumor cell types, DIFs can act as mitochondrial uncouplers, decreasing the mitochondrial membrane potential, impairing mitochondrial function, inducing reactive oxygen species (ROS) production, promoting mitochondrial fission, and ultimately triggering a caspase-independent, non-canonical form of cell death [24,25,30,31]. Although DIF-1 induces stalk cell differentiation (i.e., autophagic cell death) in *D. discoideum* [8], the detailed mechanisms underlying the action of DIFs in mammalian cells, particularly their relationship with autophagy, remain poorly understood [9].

Previous studies established anti-proliferative, mitochondrial, and signaling effects of DIF derivatives, but whether these compounds modulate autophagic flux in mammalian cells and how this response is associated with broader cellular stress pathways have remained insufficiently characterized. In the present study, we screened a panel of DIF derivatives using a mammalian autophagy-flux reporter and identified Bu-DIF-3 and CP-DIF-3 as active compounds. We subsequently examined their effects on autophagy-related signaling and cellular stress responses by combining reporter and biochemical analyses with transcriptomic, mitochondrial, redox, labile Fe^2+^, and MTT-based metabolic-activity measurements. The study therefore extends prior DIF research by linking DIF-3 derivative-induced autophagic flux to mitochondrial dysfunction and oxidative and iron-associated stress, while also revealing quantitative differences between two structurally related DIF-3 derivatives in the assays in which they were directly compared.

## 2. Results

### 2.1. Effects of DIF Compounds on Autophagy in HeLa Cells

**Figure 1:**
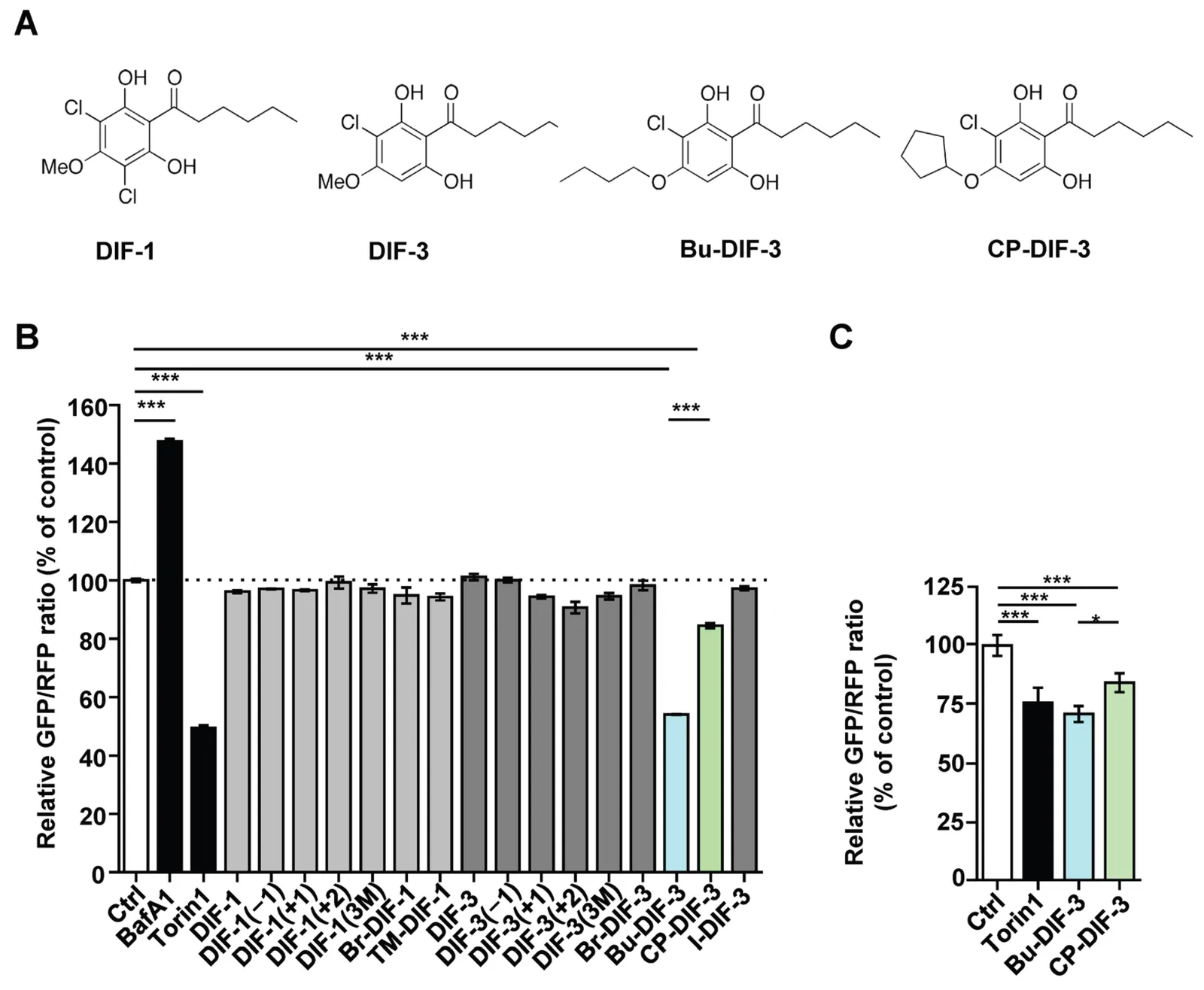
Screening of DIF derivatives for autophagic flux enhancement. (**A**) Chemical structures of DIF-1, DIF-3, Bu-DIF-3, and CP-DIF-3. (**B**) HeLa-GFP-LC3-RFP-LC3ΔG cells were treated with vehicle, DIF compounds (5 µM), Torin1 (1 µM), or BafA1 (200 nM) for 24 h. GFP/RFP ratios were normalized to the matched vehicle control. (**C**) HAP1-GFP-LC3-RFP cells were treated with vehicle, Torin1, Bu-DIF-3, or CP-DIF-3 for 24 h. Data in (**B**,**C**) are presented as mean ± SEM from three independent experiments (*n* = 3), with technical wells averaged within each experiment. Each panel was analyzed using one-way ANOVA followed by Tukey’s multiple-comparison test. * *p* < 0.05, *** *p* < 0.001.

To identify *Dictyostelium* DIFs and derivatives capable of regulating autophagy, we screened a series of DIF-related compounds using HeLa cells stably expressing the GFP-LC3-RFP-LC3ΔG probe [32]. The chemical structures and abbreviations of all screened compounds are provided in Supplementary Figure S2. At 5 µM, DIF-3 did not significantly alter the GFP/RFP ratio, whereas Bu-DIF-3 and CP-DIF-3 significantly reduced the ratio relative to vehicle (Figure 1B), consistent with enhanced reporter-defined autophagic flux. The reporter responses to Bu-DIF-3 and CP-DIF-3 were also observed in HAP1-GFP-LC3-RFP cells (Figure 1C). These results indicate that modification of the DIF-3 methoxy substituent can influence the reporter response under the tested conditions.

To verify the expected direction and time dependence of the reporter response, we additionally examined Torin1, BafA1, Bu-DIF-3, and CP-DIF-3 over 4–24 h. Torin1 reduced the GFP/RFP ratio, whereas BafA1 produced a time-dependent increase in the ratio, consistent with enhancement and blockade of autophagic flux, respectively (Figure S1A). Bu-DIF-3 and CP-DIF-3 reduced the ratio after 24 h. Concentration-response analysis further showed a larger reduction with Bu-DIF-3 than with CP-DIF-3 under the tested conditions (Figure S1B).

### 2.2. Bu-DIF-3 and CP-DIF-3 Inhibit mTORC1 Signaling and Activate AMPK

**Figure 2:**
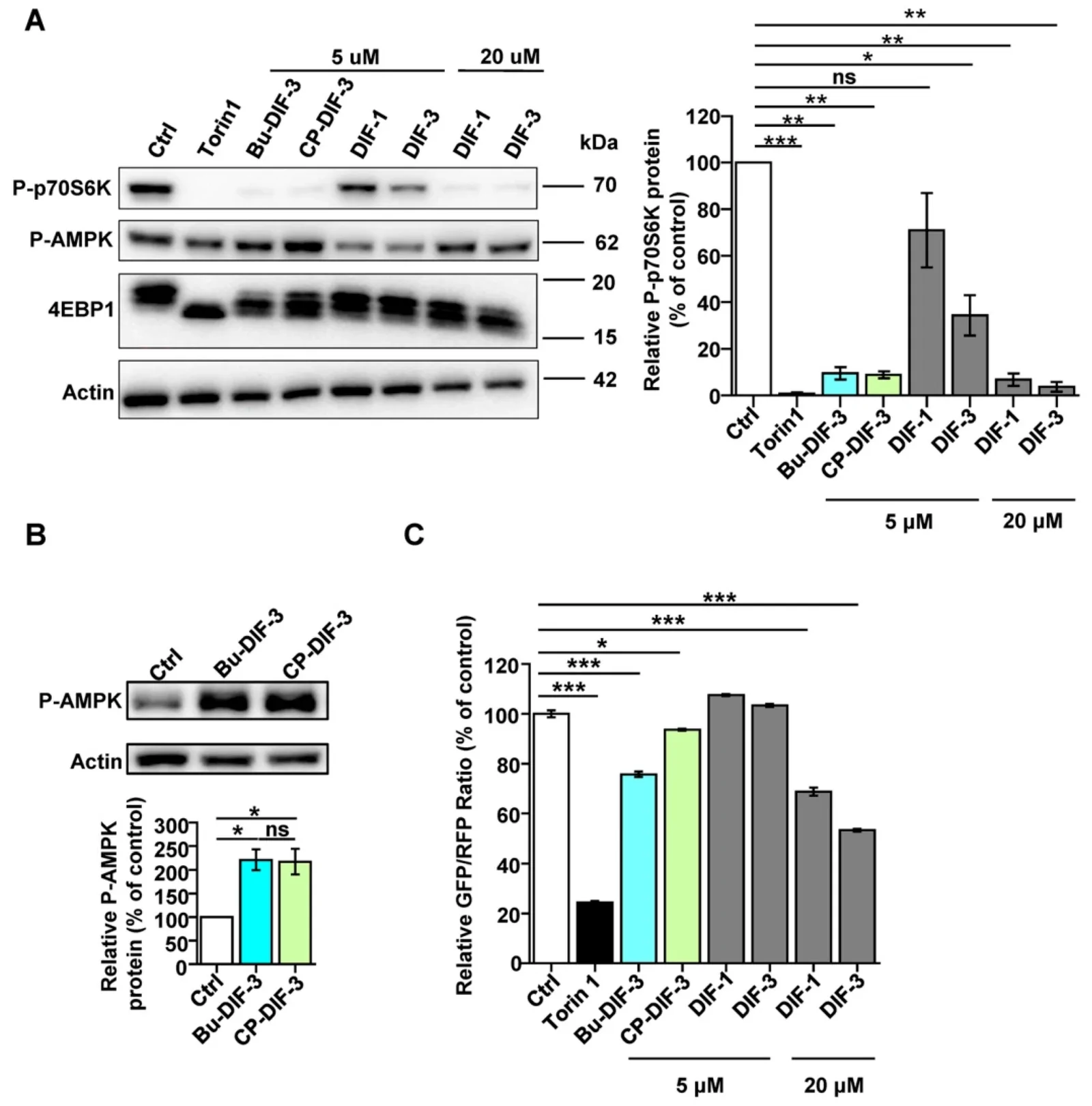
Effects of Bu-DIF-3 and CP-DIF-3 on mTORC1/AMPK signaling and reporter-defined autophagic flux. (**A**) HeLa-WT cells were treated for 24 h with Torin1 or DIF compounds at the indicated concentrations. Representative immunoblots and densitometric quantification of phospho-p70S6K are shown. Phospho-p70S6K was normalized to actin and expressed relative to the vehicle control. The 4EBP1 electrophoretic mobility pattern was evaluated qualitatively because its phosphorylation-dependent forms resolved as multiple bands. (**B**) Phospho-AMPK normalized to actin in HAP1-WT cells after 24 h treatment. (**C**) GFP/RFP ratios in HeLa reporter cells after 24 h treatment, normalized to vehicle. Data in quantified panels are mean ± SEM from three independent experiments (*n* = 3). Each panel was analyzed using one-way ANOVA followed by Tukey’s multiple-comparison test. * *p* < 0.05, ** *p* < 0.01, *** *p* < 0.001; ns, not significant.

To examine signaling changes associated with the reporter response, we analyzed mTORC1 and AMPK readouts (Figure 2). Quantification from three independent experiments showed that phospho-p70S6K, normalized to actin and expressed relative to vehicle, was significantly reduced by Torin1, Bu-DIF-3, and CP-DIF-3 at 5 µM; DIF-1 and DIF-3 had weaker effects at 5 µM and greater effects at 20 µM (Figure 2A). Torin1, Bu-DIF-3, and CP-DIF-3 also shifted 4EBP1 toward faster-migrating, hypophosphorylated forms. Bu-DIF-3 and CP-DIF-3 increased phospho-AMPK relative to actin in HAP1 cells (Figure 2B) and reduced the GFP/RFP ratio at 5 µM (Figure 2C). These parallel observations support an association among the reporter response, AMPK phosphorylation, and mTORC1 inhibition, but do not establish AMPK dependence or causal order.

### 2.3. Transcriptomic Analysis Identifies Autophagy- and Redox-Related Responses to Bu-DIF-3

**Figure 3:**
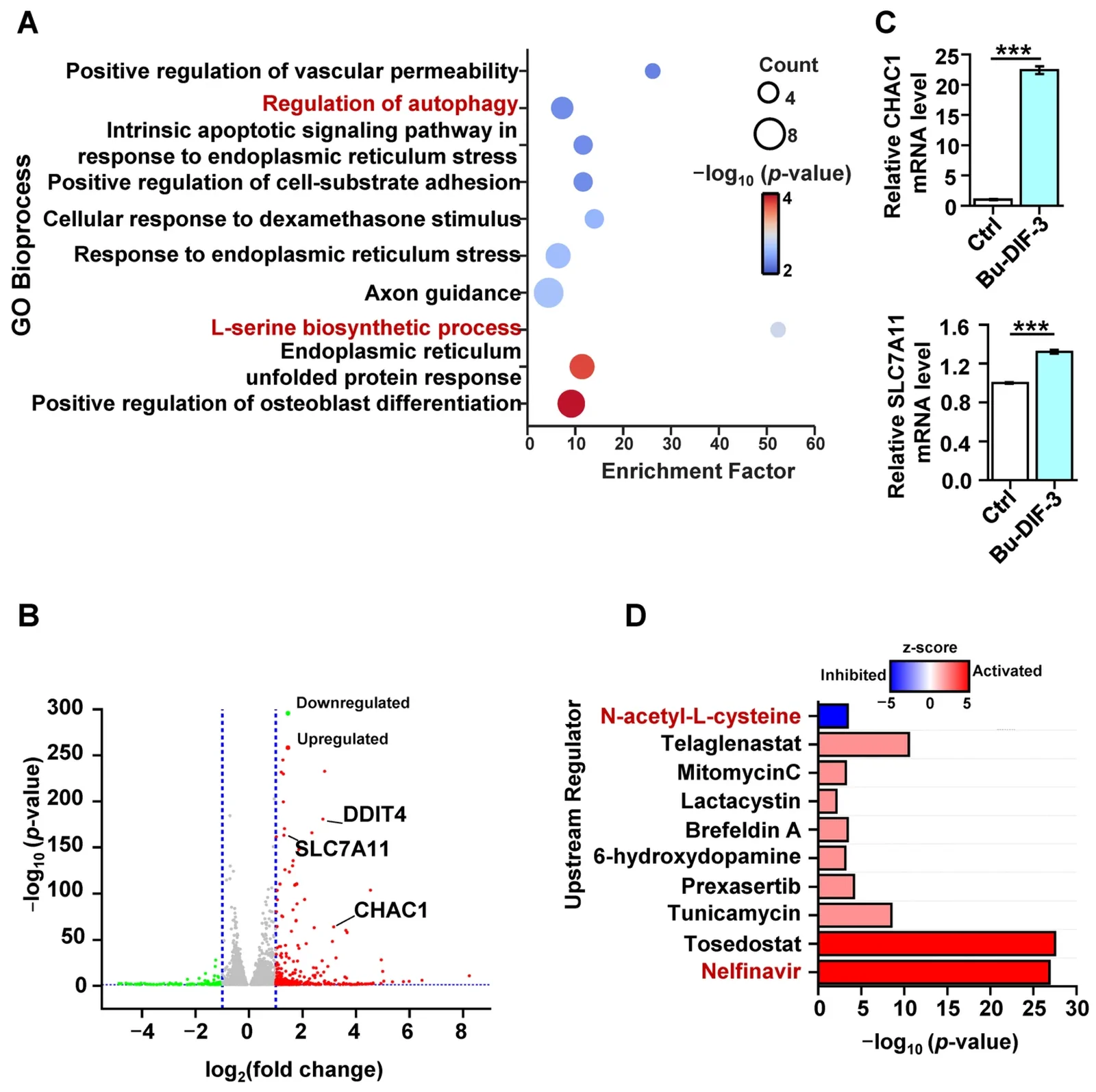
Transcriptomic analysis of Bu-DIF-3-treated HAP1-WT cells. HAP1-WT cells were treated with vehicle or Bu-DIF-3 (5 µM, 24 h); RNA-seq was performed using three independent biological replicates per condition. DEGs were defined as |log_2_FC| > 1 and padj < 0.05. (**A**) Exploratory GO biological process enrichment. (**B**) Volcano plot. (**C**) qRT-PCR validation of *CHAC1* and *SLC7A11*, normalized to GAPDH and vehicle. (**D**) IPA-predicted upstream regulators. qRT-PCR data are presented as mean ± SEM from three independent experiments (*n* = 3). Vehicle- and Bu-DIF-3-treated groups were compared separately for each gene using an unpaired two-tailed Student’s *t*-test; *** *p* < 0.001.

Although Bu-DIF-3 and CP-DIF-3 enhanced autophagic flux and modulated mTORC1 and AMPK signaling, these findings alone were insufficient to fully explain their cellular effects. Because Bu-DIF-3 enhanced autophagic flux, we performed RNA-sequencing analysis to characterize the broader cellular response to Bu-DIF-3 and identify additional pathways associated with its cellular effects. Gene Ontology (GO) enrichment analysis of the differentially expressed genes (DEGs) revealed several significantly enriched biological processes, including the regulation of autophagy and the L-serine biosynthetic process (Figure 3A). These findings indicate that Bu-DIF-3 treatment was associated with transcriptional changes in biological processes related to autophagy and L-serine biosynthesis.

We next examined the individual DEGs altered by Bu-DIF-3 treatment using a volcano plot (Figure 3B). Among the genes with markedly upregulated expression were *CHAC1*, *SLC7A11*, and *DDIT4*. We focused on *glutathione-specific γ-glutamylcyclotransferase 1* (*CHAC1*), which contributes to GSH depletion, and *solute carrier family 7 member 11* (*SLC7A11*), a key subunit of system Xc^−^ responsible for cystine uptake [33,34,35,36]. The upregulation of these genes suggests that Bu-DIF-3 induces a stress-responsive transcriptional program associated with GSH metabolic remodeling. The expression of *DDIT4*, a stress-responsive negative regulator of mTORC1, was also markedly upregulated by Bu-DIF-3. *DDIT4* is induced by cellular stresses, including hypoxia, energy depletion, oxidative stress, and ER stress [37], and suppresses mTORC1 signaling through the TSC1/TSC2 complex [38]. Thus, its induction may contribute to the inhibition of mTORC1 signaling observed following Bu-DIF-3 treatment. The induction of *CHAC1* and *SLC7A11* was further examined by quantitative RT-PCR. Bu-DIF-3 markedly increased *CHAC1* mRNA expression and produced a modest but significant increase in *SLC7A11* expression, confirming the RNA-sequencing results (Figure 3C).

To further characterize the transcriptional response to Bu-DIF-3, we performed ingenuity pathway analysis (IPA). Among the predicted upstream regulators, nelfinavir showed the most significant activation profile (Figure 3D). By contrast, N-acetyl-L-cysteine (NAC) was predicted to be inhibited, suggesting that the Bu-DIF-3-induced gene expression profile was opposite to that associated with the antioxidant effects of NAC. These predictions, together with the induction of CHAC1 and SLC7A11, are consistent with transcriptional responses associated with cellular stress and redox homeostasis. However, the IPA predictions are hypothesis-generating and do not establish a mechanistic role for nelfinavir or NAC in the response to Bu-DIF-3.

### 2.4. CP-DIF-3 Induces Transcriptional Responses Associated with Amino Acid Metabolism and Cellular Stress

**Figure 4:**
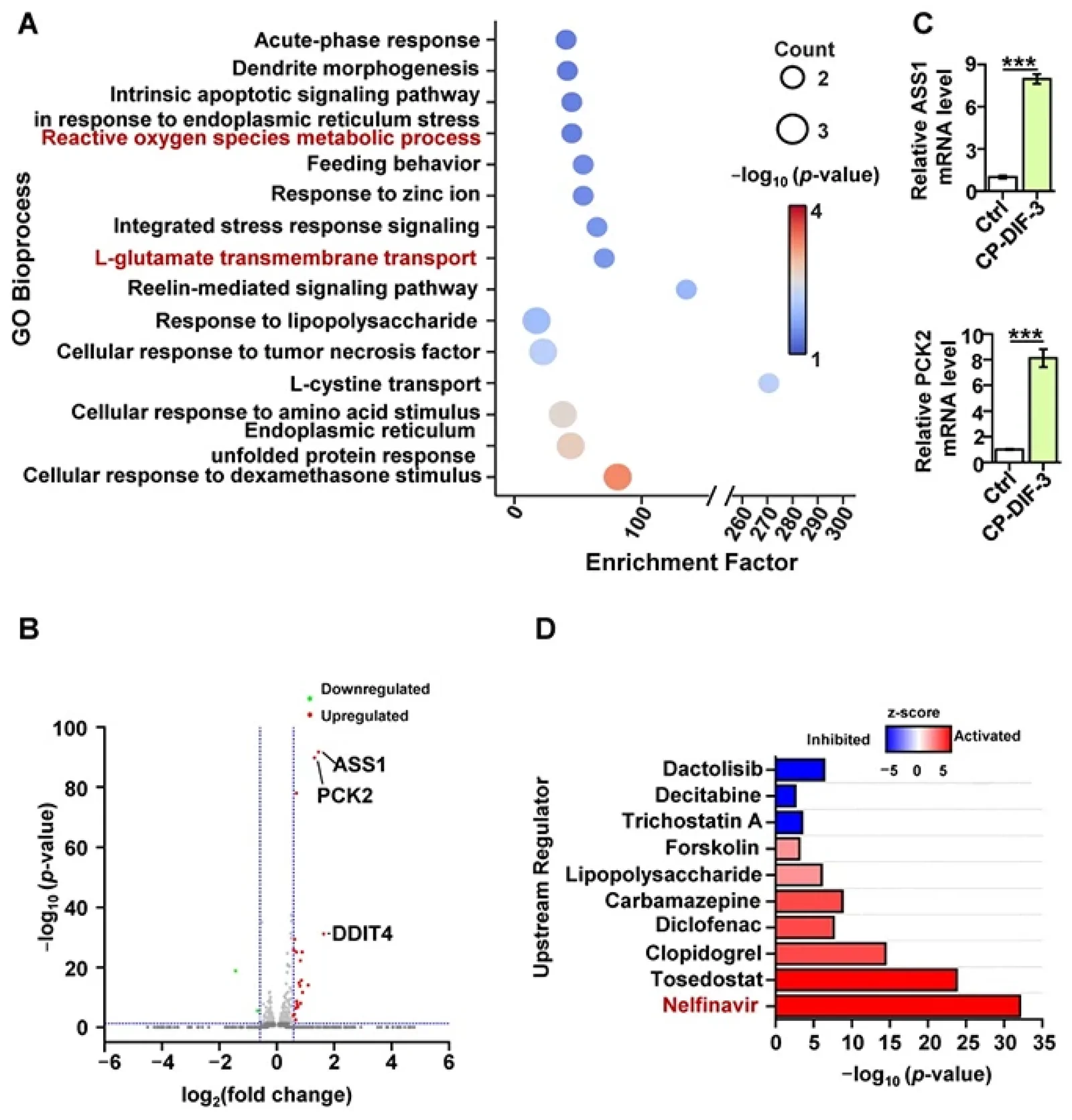
Transcriptomic analysis of CP-DIF-3-treated HAP1-WT cells. HAP1-WT cells were treated with vehicle or CP-DIF-3 (5 µM, 24 h); RNA-seq used three independent biological replicates per condition. For exploratory DEG-based enrichment, |log_2_FC| > 0.585 and padj < 0.05 were used; these DEG-based results were not used for quantitative pathway-level comparisons between the compounds; gene-level analyses using a common fold-change threshold and threshold-independent enrichment analyses are presented in Supplementary Figures S3 and S4. (**A**) GO biological process enrichment. (**B**) Volcano plot. (**C**) qRT-PCR validation of *ASS1* and *PCK2*, normalized to GAPDH and vehicle. (**D**) IPA-predicted upstream regulators. qRT-PCR data are presented as mean ± SEM from three independent experiments (*n* = 3). Vehicle- and CP-DIF-3-treated groups were compared separately for each gene using an unpaired two-tailed Student’s *t*-test; *** *p* < 0.001.

We next examined the CP-DIF-3-dependent transcriptome. Exploratory GO enrichment analysis of CP-DIF-3-treated cells revealed several significantly enriched biological processes related to the integrated stress response and redox and amino acid metabolism, including “integrated stress response signaling,” “reactive oxygen species (ROS) metabolic process,” “L-cystine transport,” “L-glutamate transmembrane transport,” and “cellular response to amino acid stimulus” (Figure 4A). Volcano plot analysis identified *argininosuccinate synthase 1* (*ASS1*), *phosphoenolpyruvate carboxykinase 2* (*PCK2*), and *DDIT4* among the genes with upregulated expression following CP-DIF-3 treatment (Figure 4B). The induction of *ASS1* and *PCK2* was subsequently confirmed by quantitative RT-PCR (Figure 4C), supporting the RNA-sequencing results [39,40].

IPA upstream regulator analysis predicted activation of a nelfinavir-associated transcriptional signature and inhibition of a dactolisib-associated signature (Figure 4D). A similar predicted nelfinavir-associated signature was observed in the Bu-DIF-3 dataset, suggesting a partial overlap between the transcriptional responses to Bu-DIF-3 and CP-DIF-3. Collectively, these results suggest that CP-DIF-3 treatment is associated with transcriptional changes in pathways related to amino acid metabolism and redox regulation, although these predictive analyses do not establish the underlying regulatory mechanisms.

To compare the transcriptional responses using a consistent gene-level criterion, volcano plots for both contrasts were generated using the same fold-change threshold (|log_2_FC| > 1). Under this common criterion, Bu-DIF-3 showed a broader set of genes exceeding the cutoff than CP-DIF-3; these results were interpreted descriptively and were not used to compare pathway-enrichment strength quantitatively (Figure S3A,B). Principal-component analysis of the top 1000 variable genes, which did not rely on a DEG cutoff, showed clear separation among the control, Bu-DIF-3, and CP-DIF-3 samples (Figure S3C).

We further performed threshold-independent pre-ranked gene set enrichment analysis using the complete DESeq2 Wald-statistic rankings. Both treatments showed positive enrichment of unfolded-protein-response, hypoxia, heme-metabolism, p53, regulation-of-autophagy, and amino acid-response gene sets, together with negative enrichment of E2F, G2M, and MYC target programs. In contrast, mTORC1 signaling and oxidative phosphorylation showed different enrichment directions between the two compounds (Supplementary Figures S3D,E and S4 and Data S1).

Collectively, these results suggest that CP-DIF-3 affects pathways associated with amino acid metabolism and redox regulation and that Bu-DIF-3 and CP-DIF-3 induce partially overlapping but distinct transcriptional stress responses.

### 2.5. Effects of Bu-DIF-3 and CP-DIF-3 on MMP and ROS Production

**Figure 5:**
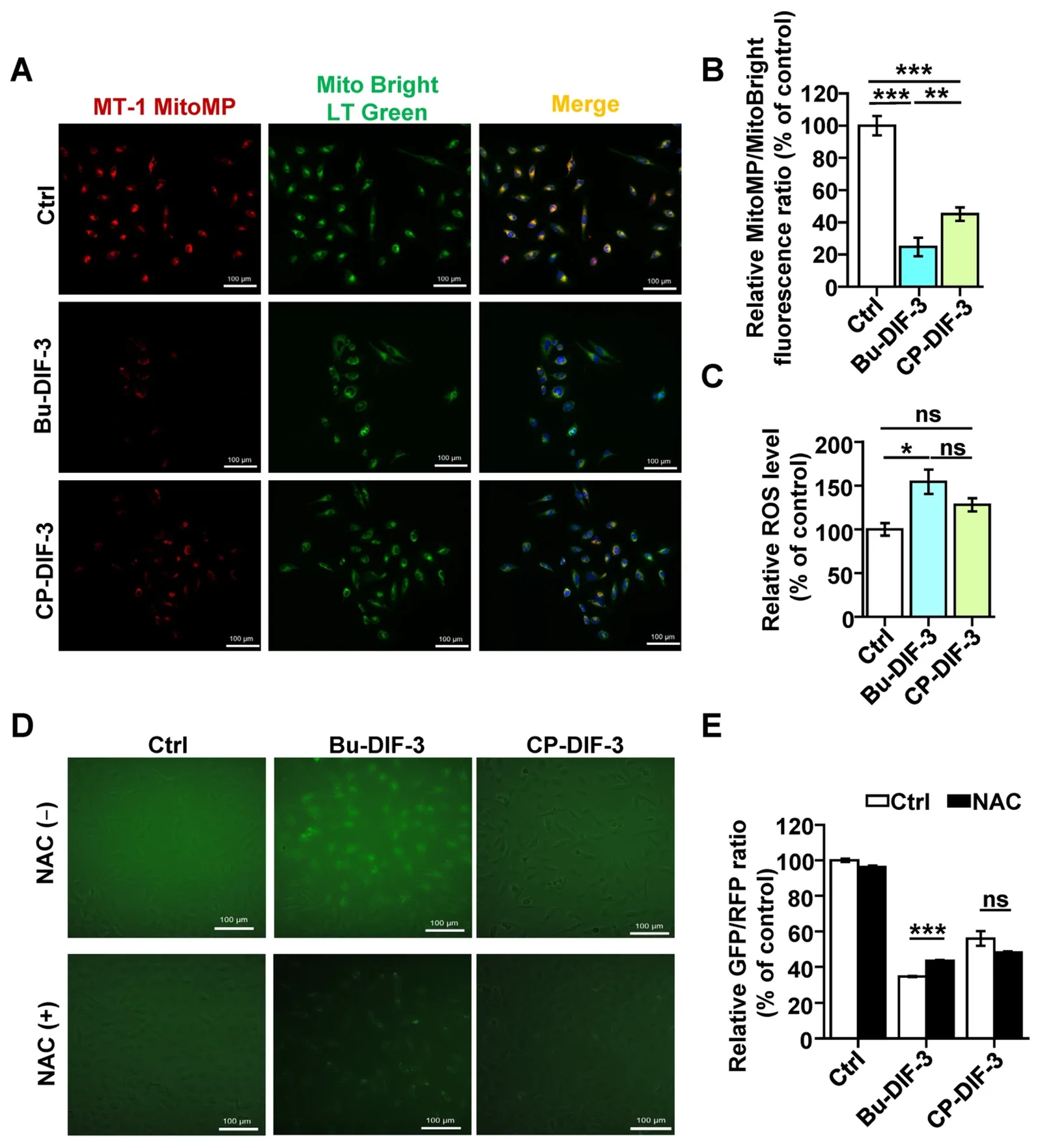
Effects of Bu-DIF-3 and CP-DIF-3 on mitochondrial function and ROS production. (**A**) Representative fluorescence images of HeLa-WT cells stained with MT-1 MitoMP and MitoBright LT Green after treatment with Bu-DIF-3 or CP-DIF-3 (5 µM, 24 h). Scale bars, 100 µm. (**B**) Quantification of the MT-1 MitoMP/MitoBright LT Green fluorescence intensity ratio; both compounds significantly reduced the membrane potential-associated signal. Fluorescence was quantified from multiple randomly acquired 20× fields in three independent experiments, with more than three fields and more than 100 cells analyzed in total. Measurements from multiple fields were averaged within each experiment. (**C**) CellROX Green fluorescence in HeLa-WT cells after treatment with Bu-DIF-3 or CP-DIF-3 (5 µM, 24 h). Bu-DIF-3 significantly increased the CellROX Green signal, whereas the increase following CP-DIF-3 treatment did not reach statistical significance. (**D**) Representative CellROX Green fluorescence images in the absence or presence of 5 mM NAC (10 µM Bu-DIF-3 or CP-DIF-3, 24 h). Scale bars, 100 µm. (**E**) GFP/RFP reporter ratio as an indicator of autophagic flux in HeLa-GFP-LC3-RFP-LC3ΔG cells treated with Bu-DIF-3 or CP-DIF-3 (10 µM, 24 h) in the absence or presence of NAC (5 mM). NAC partially attenuated the reduction in the GFP/RFP ratio induced by Bu-DIF-3, whereas it did not significantly affect the reduction induced by CP-DIF-3. Data are presented as the mean ± SEM from three independent experiments (*n* = 3). (**B**,**C**) were analyzed separately using one-way ANOVA followed by Tukey’s multiple-comparison test; (**E**) was analyzed using two-way ANOVA followed by Tukey’s multiple-comparison test. Image quantification was performed without blinding. * *p* < 0.05, ** *p* < 0.01, *** *p* < 0.001; ns, not significant.

RNA-seq analysis revealed that Bu-DIF-3 and CP-DIF-3 treatments were associated with transcriptional responses related to cellular stress and redox homeostasis. Considering that mitochondrial dysfunction can promote ROS generation and disrupt redox homeostasis, we examined whether these transcriptional responses were accompanied by alterations in MMP (ΔΨm). Cells were stained with MT-1 MitoMP, a membrane potential-dependent mitochondrial probe, together with MitoBright LT Green, which labels mitochondria independently of membrane potential. The fluorescence intensity ratio of MitoMP to MitoBright was used as an indicator of ΔΨm. In the control cells, the MitoMP signal colocalized with the mitochondrial network labeled by MitoBright (Figure 5A). By contrast, Bu-DIF-3 and CP-DIF-3 significantly reduced the MitoMP signal, whereas the MitoBright signal remained comparable to that in the control cells. Quantitative analysis confirmed that both compounds significantly reduced the MitoMP/MitoBright ratio (Figure 5B). These results indicate that Bu-DIF-3 and CP-DIF-3 induce mitochondrial membrane depolarization.

Intracellular ROS levels were measured using CellROX Green staining. Bu-DIF-3 significantly increased the CellROX Green signal compared with the control (Figure 5C). Although CP-DIF-3 tended to increase the CellROX Green signal, the change did not reach statistical significance. To examine whether modulation of the cellular redox state was associated with the reporter response, cells were treated with the antioxidant NAC. NAC reduced the CellROX Green signal induced by Bu-DIF-3 (Figure 5D) and partially attenuated the Bu-DIF-3-induced decrease in the GFP/RFP ratio (Figure 5E). By contrast, NAC did not significantly alter the CP-DIF-3-induced change in the GFP/RFP ratio. These findings are consistent with an association between NAC-sensitive redox changes and the Bu-DIF-3-induced reporter response.

### 2.6. Bu-DIF-3 Increases the FerroOrange-Detectable Labile Fe^2+^ Signal and Reduces MTT-Derived Cellular Metabolic Activity

**Figure 6:**
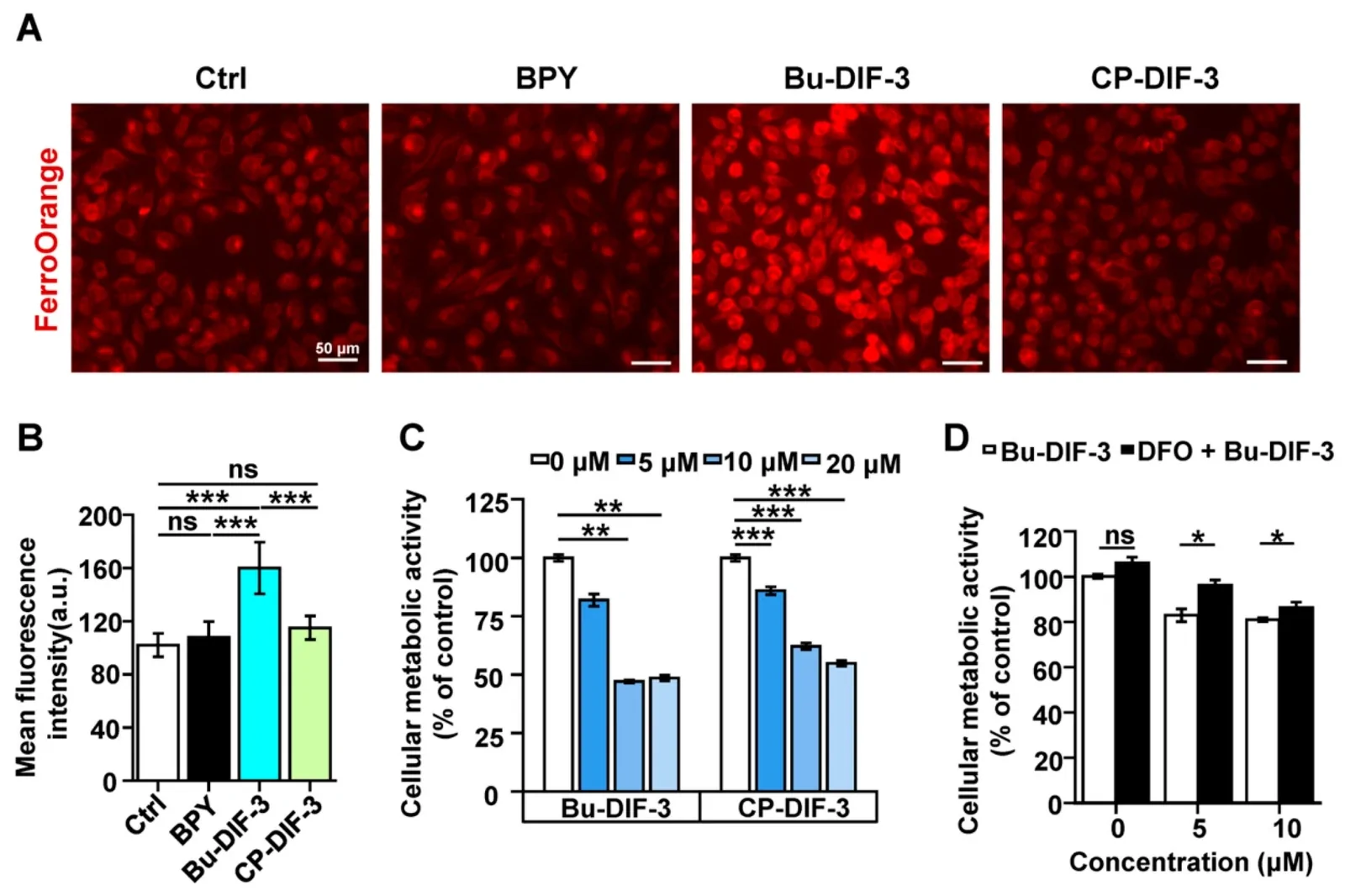
Effects of Bu-DIF-3 and CP-DIF-3 on the FerroOrange-detectable labile Fe^2+^ signal and MTT-derived cellular metabolic activity. (**A**) Representative FerroOrange images after vehicle, BPY (100 µM), Bu-DIF-3 (5 µM), or CP-DIF-3 (5 µM) treatment for 24 h. Scale bars, 50 µm. (**B**) FerroOrange intensity normalized to vehicle. For panel B, fluorescence was quantified from multiple randomly acquired 20× fields in three independent experiments, with more than three fields and more than 100 cells analyzed in total. Field measurements were averaged within each experiment. The mean of the three vehicle-control values was used as a common reference representing 100%. Each experiment-level value was divided by this reference and multiplied by 100, and the mean ± SEM was calculated from the three normalized values in each group. BPY was included as an iron-chelator treatment but did not significantly reduce the basal signal under this protocol and was not interpreted as a validated negative control. (**C**) MTT-derived cellular metabolic activity after 24 h treatment at the indicated concentrations. (**D**) Effect of DFO (25 µM) on the Bu-DIF-3-induced MTT response after 6 h. For (**C**,**D**), 24 h and 6 h indicate the treatment periods before MTT reagent addition. After treatment, the compound-containing medium was removed, the cells were washed once with PBS, and MTT was added in fresh drug-free medium for a further 4 h. Data for (**B**–**D**) are presented as the mean ± SEM from three independent experiments (*n* = 3). For (**C**,**D**), each experiment included five technical wells per condition, which were averaged before summary statistics and statistical analysis. Data are mean ± SEM; *n* = 3 independent experiments for (**B**) and *n* = 5 for (**C**,**D**). * *p* < 0.05, ** *p* < 0.01, *** *p* < 0.001; ns, not significant. (**B**) was analyzed using one-way ANOVA followed by Tukey’s multiple-comparison test; (**C**,**D**) were analyzed using two-way ANOVA followed by Tukey’s multiple-comparison test. Image quantification was not blinded.

The transcriptional induction of CHAC1 and SLC7A11, together with the mitochondrial depolarization and increased CellROX fluorescence induced by Bu-DIF-3, suggested a possible disruption of cellular iron and redox homeostasis [35]. We therefore examined intracellular labile ferrous iron (Fe^2+^) levels using the fluorescent probe FerroOrange (Figure 6A). Treatment with 5 μM Bu-DIF-3 significantly increased the FerroOrange fluorescence signal by approximately 1.5- to 1.7-fold compared with the control (Figure 6A,B), indicating an increase in the FerroOrange-detectable intracellular labile Fe^2+^ signal. By contrast, 5 μM CP-DIF-3 did not significantly increase FerroOrange fluorescence under the same conditions. BPY (100 µM, 24 h) did not significantly reduce basal FerroOrange fluorescence and therefore did not establish a validated chelation control under the detection conditions.

The effects of Bu-DIF-3 and CP-DIF-3 on MTT-derived cellular metabolic activity were examined over a concentration range (Figure 6C). Both compounds reduced the MTT signal following a 24 h treatment period before MTT reagent addition. DFO partially attenuated the reduction produced by 5 or 10 µM Bu-DIF-3 following a 6 h treatment period before MTT reagent addition (Figure 6D), supporting an iron-sensitive component without establishing that iron directly caused oxidative damage or cell death. In a co-treatment experiment, MRT68921 (1 µM) did not restore the MTT signal reduced by Bu-DIF-3 and appeared to reduce it further under the tested conditions (Figure S1C).

## 3. Discussion

### 3.1. Autophagy-Inducing Activity of DIF Derivatives

Autophagy is a highly conserved intracellular catabolic process that maintains cellular homeostasis by recycling cellular constituents. Because autophagy can contribute to tumor suppression or the induction of cell death under certain conditions [5], modulation of this pathway has emerged as a promising strategy for cancer therapy.

DIF-1 and DIF-3 were originally identified as inducers of stalk cell differentiation, a form of autophagic cell death, in *D. discoideum* [6,7,8]. Interestingly, DIFs and their derivatives exhibit diverse biological activities in mammalian cells, including anti-tumor, glucose metabolism-promoting, and immunoregulatory effects, as well as anti-bacterial, anti-trypanosomal, and anti-malarial activities [9,12,13,14,15,18,19,20,21,22]. Considerable effort has been devoted to elucidating the anti-tumor activities of DIF derivatives and their underlying mechanisms of action. However, the molecular mechanisms by which DIF derivatives act in tumor cells, particularly their relationship with autophagy, remain poorly understood [9].

Using a fluorescence-based autophagic flux reporter in HeLa cells, we screened a library of DIF derivatives and identified Bu-DIF-3 and CP-DIF-3 as compounds that reduced the GFP/RFP ratio. These findings suggest that modification of the DIF-3 methoxy substituent influences the reporter response. Bu-DIF-3 and CP-DIF-3 have previously shown anti-proliferative and anti-migratory effects in several tumor cell models [13,14,31], but the present study does not establish anticancer efficacy.

### 3.2. Effects of DIF Derivatives on the AMPK–mTORC1 Pathway

At 5 µM for 24 h, Bu-DIF-3 and CP-DIF-3 produced several shared cellular responses, including mitochondrial depolarization, increased AMPK phosphorylation, reduced mTORC1 signaling, and enhanced autophagic flux. Under these matched conditions, only Bu-DIF-3 significantly increased the CellROX and FerroOrange fluorescence signals (Supplementary Table S2). The observed pattern is consistent with a working model in which mitochondrial stress, AMPK phosphorylation, and reduced mTORC1 signaling accompany reporter-defined autophagic flux [41,42]. This interpretation is consistent with previous studies showing that DIF-1 activates AMPK, inhibits mTORC1–S6K signaling, and suppresses tumor growth and metastasis [17], whereas DIF-3 induces mitochondrial depolarization, ROS production, and DRP1-dependent mitochondrial fission, leading to caspase-independent cell death [23]. In addition, DIF compounds penetrate cellular membranes, localize predominantly to mitochondria, and act as mitochondrial uncouplers in both *D. discoideum* and mammalian cells [25,31]. Collectively, these previous reports and our findings suggest that mitochondria are plausible early cellular targets of lipophilic DIF derivatives. However, AMPK dependency and the temporal relationships among mitochondrial dysfunction, AMPK activation, mTORC1 inhibition, and autophagy were not directly examined. Thus, the proposed mitochondrial stress–AMPK–mTORC1–autophagy axis should be regarded as a working model for future studies.

Transcriptomic analyses further indicated that treatment with 5 μM Bu-DIF-3 or CP-DIF-3 elicited overlapping but distinct cellular stress responses. Threshold-independent pre-ranked GSEA showed shared enrichment of stress-related gene sets and negative enrichment of E2F, G2M, and MYC target programs. In contrast, mTORC1 signaling and oxidative phosphorylation showed different enrichment directions between the two compounds. Moreover, IPA predicted activation of nelfinavir in both datasets, suggesting partial similarity between their transcriptional signatures and those associated with nelfinavir. Given that nelfinavir suppresses mTORC1 signaling [43], this prediction is consistent with our biochemical finding that Bu-DIF-3 and CP-DIF-3 inhibit the mTORC1 pathway.

### 3.3. Effects of DIF Derivatives on Intracellular Labile Fe^2+^ Signal

A key difference between the two compounds was that, at 5 µM, Bu-DIF-3 but not CP-DIF-3, increased the intracellular labile Fe^2+^ signal. Moreover, DFO partially attenuated the Bu-DIF-3-induced reduction in MTT-derived cellular metabolic activity, supporting a possible iron-sensitive component of this response without establishing that iron directly caused oxidative injury or cell death. Labile Fe^2+^ may amplify oxidative stress through iron-dependent ROS generation, forming a feed-forward loop among mitochondrial dysfunction, ROS accumulation, and iron dysregulation [44]. The source of increased labile Fe^2+^ signal remains unclear but may involve altered iron uptake, storage, trafficking, lysosomal iron processing, or NCOA4-dependent ferritinophagy [45,46]. As autophagy-mediated ferritin degradation can increase intracellular iron and sensitize cells to oxidative injury [46,47], an autophagy–iron stress axis is an attractive hypothesis for the effects of Bu-DIF-3, although it remains to be tested directly. However, BPY did not significantly reduce basal FerroOrange fluorescence. BPY was absent from the staining solution, and chelator-sensitive suppression of the signal was not demonstrated under this protocol. The FerroOrange result should therefore be interpreted with this unresolved control limitation.

Treatment with MRT68921 did not restore the Bu-DIF-3-reduced MTT signal and further decreased it under selected conditions. These observations raise the possibility that the autophagic response may have an adaptive role; however, this remains a hypothesis for future investigation. Autophagic flux was not assessed under the co-treatment conditions, so the additional decrease in MTT signal cannot be attributed specifically to inhibition of an adaptive autophagic response. Moreover, the MTT endpoint reflects cellular metabolic activity rather than cell survival. Whether autophagy protects cells against the Bu-DIF-3-induced reduction in metabolic activity therefore remains unresolved. Genetic approaches using cells deficient in autophagy-related genes, combined with viability assays that do not depend on cellular reductase activity, would be required to address this question more directly.

Bu-DIF-3 increases lipid body formation in HeLa cells, suggesting altered lipid metabolism secondary to mitochondrial dysfunction [25]. Because lipid droplets regulate fatty acid storage and susceptibility to lipid peroxidation [48], these changes may influence iron-dependent oxidative injury and reduced cellular metabolic activity, although their functional significance remains unknown. Taken together, our findings indicate an association among enhanced autophagic flux, mitochondrial dysfunction, ROS accumulation, alteration of the labile Fe^2+^ signal, altered lipid metabolism, and reduced cellular metabolic activity. However, the present data do not establish the temporal sequence or causal relationships among these cellular responses, including whether autophagy is protective, detrimental, or incidental. Labile iron may contribute to oxidative stress and reduced cellular metabolic activity, although this relationship was not established directly. The differential NAC response may reflect differences in redox sensitivity, ROS burden, or other NAC-responsive cellular processes; however, the present data do not distinguish among these possibilities. Only one ROS-modulating reagent was examined; a mechanistically distinct intervention will be required to establish the direction and specificity of the ROS–autophagy relationship.

Several limitations of this study should be acknowledged. First, although mitochondrial depolarization, AMPK activation, mTORC1 inhibition, ROS accumulation, increased labile Fe^2+^ signal, and enhanced autophagic flux were observed, the other mechanistic readouts were not examined across matched concentrations and time points, and their temporal sequence and causal relationships were not established. Pharmacological or genetic perturbation of AMPK will be required to determine whether AMPK activation is necessary for the autophagic response. Second, a less stringent fold-change threshold was applied for exploratory pathway analysis because CP-DIF-3 yielded fewer DEGs than Bu-DIF-3 under identical criteria. Consequently, the difference in DEG numbers should not be interpreted as a quantitative measure of transcriptional response, and the pathway assignments for CP-DIF-3 require independent validation. Third, the MTT assay primarily reflects metabolic activity and does not by itself distinguish cytostatic from cytotoxic effects or identify the mode of cell death. Finally, although Bu-DIF-3 increased the intracellular labile Fe^2+^ signal and DFO partially restored cellular metabolic activity, we did not directly examine lipid peroxidation, GPX4 dependence, or rescue by ferroptosis-specific inhibitors such as ferrostatin-1 or liproxstatin-1. Thus, our findings are consistent with the involvement of iron-dependent oxidative injury, but are insufficient to establish canonical ferroptosis or any mode of cell death. Consistent with this interpretation, induction of *SLC7A11* is compatible with a compensatory antioxidant response rather than a straightforward pro-ferroptotic signal [49]. Further studies incorporating direct measurements of lipid peroxidation together with defined genetic and pharmacological manipulation of ferroptosis pathways will be required to determine whether ferroptosis contributes to the Bu-DIF-3 response.

## 4. Materials and Methods

### 4.1. Cell Lines and Cell Culture

Human cervical cancer HeLa (wild-type, WT, RCB0007) cells were obtained from the RIKEN BioResource Research Center (RIKEN BRC, Tsukuba, Ibaraki, Japan), and human near-haploid HAP1 cells (WT) were obtained from Horizon Discovery (Cambridge, UK). The pMRX-IP-GFP-LC3-RFP-LC3ΔG plasmid was provided by Professor Noboru Mizushima (the University of Tokyo, Japan). Stable HeLa cells expressing GFP-LC3-RFP-LC3ΔG and HAP1 cells expressing GFP–LC3–RFP, which were previously established in our laboratory, were cultured as described previously [32,50]. HeLa and HAP1 cells were cultured in high-glucose Dulbecco’s Modified Eagle Medium (DMEM; Gibco, Waltham, MA, USA) and Iscove’s Modified Dulbecco’s Medium (Gibco), respectively, each supplemented with 10% fetal bovine serum (FBS, Gibco), 100 U/mL penicillin, and 100 µg/mL streptomycin (Gibco), at 37 °C in a humidified incubator containing 5% CO_2_.

### 4.2. Reagents and Antibodies

DIF derivatives were synthesized as previously described [13], dissolved in DMSO to prepare 10 mM stocks, stored at −20 °C, and protected from light. The screening panel comprised DIF-1, DIF-1(−1), DIF-1(+1), DIF-1(+2), DIF-1(3M), Br-DIF-1, TM-DIF-1, DIF-3, DIF-3(−1), DIF-3(+1), DIF-3(+2), DIF-3(3M), Br-DIF-3, Bu-DIF-3, CP-DIF-3, and I-DIF-3 (structures in Supplementary Figure S2). The (−1), (+1), and (+2) labels denote side-chain-length variants relative to the parent scaffold; 3M denotes the branched 3-methyl analog; Br and I denote halogen-substituted analogs; and TM, Bu, and CP denote trimethoxy-, butoxy-, and cyclopentyloxy-substituted derivatives, respectively. Bafilomycin A1 (BafA1), Torin1, N-acetylcysteine (NAC), deferoxamine (DFO), 2,2′-bipyridyl (BPY), phosphatase-inhibitor cocktail, BCA protein-assay kit, and skim milk were purchased from FUJIFILM Wako Pure Chemical Corporation (Osaka, Japan). The protease-inhibitor cocktail was obtained from Roche (Basel, Switzerland). Antibodies were anti-phospho-p70S6K (Thr389; CST #97596), anti-4EBP1 (CST #9644), anti-phospho-AMPKα (Thr172; CST #2535), and anti-pan-actin (CST #41185). HRP-conjugated secondary antibodies were obtainedfrom Jackson ImmunoResearch (West Grove, PA, USA). MRT68921 hydrochloride (Selleck Chemicals, Houston, TX, USA, Cat. No. S7949) was used as a ULK1/2 inhibitor.

### 4.3. Autophagic Flux Screening and Flow Cytometric Analysis

HeLa-GFP-LC3-RFP-LC3ΔG and HAP1-GFP-LC3-RFP cells [32] were seeded at 3 × 10^4^ cells/well in 24-well plates. After 24 h, cells were treated with vehicle, DIF derivatives, Torin1 (1 µM), or BafA1 (200 nM) under the conditions stated in each figure legend. For the supplementary time course, cells were treated for 4, 8, or 24 h; for the concentration-response experiment, Bu-DIF-3 and CP-DIF-3 were tested at 0, 1, 5, and 10 µM for 24 h. At least 5000 events were collected per sample on a Sony SA3800 spectral analyzer (Sony Corporation, Tokyo, Japan). The mean GFP/RFP fluorescence intensity ratio was calculated and normalized to the matched vehicle control. Technical wells were averaged within each experiment, and n denotes independent experiments; the reporter experiments shown in Figure 1, Figure 2 and Figure S1A,B were repeated independently three times.

### 4.4. Western Blot

HeLa or HAP1 cells were seeded at 1 × 10^5^ cells/well in 12-well plates and incubated for 24 h. Cells were lysed on ice for 10 min in lysis buffer containing 50 mM Tris-HCl (pH 7.4), 150 mM NaCl, 1 mM EDTA, 1% Triton X-100, and protease and phosphatase inhibitors. The lysates were centrifuged (15 min), and protein concentration was determined using a BCA assay. Equal amounts of protein (10 µg) were separated by SDS-PAGE and transferred onto PVDF membranes. The membranes were blocked with 5% skim milk in TBST (0.1% Tween-20) for 1 h at room temperature, washed with TBS, and then incubated overnight at 4 °C with primary antibodies. After being washed, the membranes were incubated with HRP-conjugated secondary antibodies for 1 h at room temperature and visualized using SuperSignal West Dura/Pico Extended Duration Substrate (Thermo Fisher Scientific, Waltham, MA, USA). Bands were imaged with a Fusion chemiluminescence system and quantified using FusionCapt Advance Solo 7 software (Vilber Lourmat, Collégien, France), with β-actin as an internal control [50].

### 4.5. RNA Sequencing and Bioinformatic Analysis

HAP1-WT cells were seeded at 2 × 10^5^ cells/well in 6-well plates and then treated with 5 µM Bu-DIF-3, 5 µM CP-DIF-3, or 0.1% DMSO for 24 h. Total RNA was extracted using the ReliaPrep RNA Cell Miniprep System (Promega, Madison, WI, USA). Poly(A)+ mRNA was enriched from total RNA, and sequencing libraries were prepared. Sequencing was performed on an IlluminaNovaSeq 6000 platform (Illumina, Inc., San Diego, CA, USA), generating 150-bp paired-end reads. RNA sequencing was performed using three independently prepared biological replicates per condition (vehicle control, Bu-DIF-3, and CP-DIF-3), for a total of nine libraries. Samples were not pooled, and each biological replicate was retained as an independent sample in DESeq2.

Raw reads were filtered by removing those containing adapter contamination, those with more than 10% undetermined bases, and those in which more than 50% of the bases had quality scores of Q5 or lower. Clean reads were aligned to the human reference genome (hg38) using HISAT2 v2.0.5. Gene-level read counts were generated using featureCounts v1.5.0-p3, and differential expression analysis was performed using DESeq2 v1.20.0. The resulting differential expression data were filtered, organized, and visualized using Origin 2021 (OriginLab, Northampton, MA, USA). Per-library sequencing and alignment quality-control metrics, including raw and clean read counts, error rate, Q20, Q30, GC content, overall and unique mapping rates, and gene-assignment metrics, are provided in Supplementary Table S1.

For Bu-DIF-3-treated cells, DEGs were selected using thresholds of |log_2_ fold change (log_2_FC)| > 1 and padj < 0.05. Applying the same criteria to the CP-DIF-3-treated cells identified only five DEGs, which was insufficient for a meaningful pathway enrichment analysis. Therefore, the fold-change threshold for CP-DIF-3 was relaxed to |log_2_FC| > 0.585 and padj < 0.05. The different DEG thresholds were used only for exploratory within-treatment enrichment, and no quantitative comparison of DEG numbers or DEG-based enrichment strength was made between the compounds. GO enrichment analysis was performed using the DAVID Bioinformatics Resources (https://davidbioinformatics.nih.gov/, accessed on 23 October 2025), and upstream regulator analysis was conducted using Ingenuity Pathway Analysis (QIAGEN, Redwood City, CA, USA).

For descriptive comparison under a common fold-change threshold, volcano plots for both contrasts were generated using |log_2_FC| > 1, and principal component analysis was performed using the top 1000 variable genes. For threshold-independent comparison, the count matrix was reanalyzed in R v4.4.0 using DESeq2 v1.46.0 after retaining genes with a total count of at least 10 across all samples. Genes were ranked according to the DESeq2 Wald statistic, and pre-ranked gene set enrichment analysis was performed using fgseaMultilevel in fgsea v1.32.4 with MSigDB Hallmark and C5 GO Biological Process gene sets obtained using msigdbr v26.1.1. Gene set sizes were restricted to 15–500 genes, and Benjamini–Hochberg FDR *q* < 0.05 was used as the significance criterion. Complete analysis parameters and results are provided in Supplementary Data S1.

The raw RNA-sequencing data and count matrix generated in this study are available in the NCBI Gene Expression Omnibus database under accession number GSE341456.

### 4.6. RNA Extraction and Quantitative Real-Time PCR (qPCR)

RNA extraction and treatments were performed as described in Section 4.5. cDNA was synthesized from 0.5 µg total RNA using the PrimeScript RT Reagent Kit with gDNA Eraser (Takara Bio Inc., Kusatsu, Shiga, Japan). qPCR used TB Green Premix Ex Taq II and the 2^−ΔΔCt^ method with *GAPDH* normalization. Primer sequences were: *SLC7A11* forward 5′-TCAGTTTTGCCAGGTGGCTT-3′, reverse 5′-GGATGAACAGTGGCACCTTG-3′; *CHAC1* forward 5′-TTGAAGATCATGAGGGCTGC-3′, reverse 5′-GCAAGTATTCAAGGTTGTGGC-3′; *PCK2* forward 5′-GAGAATACTGCCACACTGACC-3′, reverse 5′-CCGCTGAGAAGGAGTTACAATC-3′; *ASS1* forward 5′-TGTGCTTATAACCTGGGATGG-3′, reverse 5′-CATAGCGTCTGGGATTGGAG-3′; *GAPDH* forward 5′-ACCACAGTCCATGCCATCAC-3′, reverse 5′-TCCACCACCCTGTTGCTGTA-3′.

### 4.7. Assessment of Cellular Stress Responses

For image-based assays, acquisition and quantification procedures are described below. Quantification was not performed blinded to treatment condition.

#### 4.7.1. Detection of Intracellular ROS

Intracellular ROS levels were measured using CellROX Green Reagent (Thermo Fisher Scientific). HeLa cells were seeded at 3 × 10^4^ cells/well in 24-well plates and treated with 0.1% DMSO, 5 µM Bu-DIF-3, or 5 µM CP-DIF-3 for 24 h. The cells were washed twice with PBS and incubated with 5 µM CellROX Green at 37 °C for 30 min in the dark. After being washed, the cells were trypsinized, resuspended in phenol red-free DMEM, and then analyzed by flow cytometry to quantify fluorescence intensity [51]. For fluorescence imaging, cells were treated with vehicle or 10 µM Bu-DIF-3 or CP-DIF-3 for 24 h in the absence or presence of 5 mM NAC. For NAC co-treatment, NAC was added 30 min before the DIF derivatives without changing the medium. The cells were stained with 5 µM CellROX Green for 30 min, washed, and then visualized under a BZ-X810 fluorescence microscope (Keyence, Osaka, Japan) using a 20× objective lens.

#### 4.7.2. ROS Inhibition Assay

The antioxidant NAC was used to examine whether NAC altered Bu-DIF-3- and CP-DIF-3-induced changes in the autophagic flux reporter [52]. HeLa-GFP-LC3-RFP-LC3ΔG cells were seeded at 3 × 10^4^ cells/well in 24-well plates and incubated for 24 h. The cells were divided into six groups: control (0.1% DMSO + 1% Milli-Q water (MQ)), 5 mM NAC, 10 µM Bu-DIF-3, 10 µM CP-DIF-3, 10 µM Bu-DIF-3 + 5 mM NAC, and 10 µM CP-DIF-3 + 5 mM NAC. For NAC treatments, the cells were preincubated with NAC for 30 min before adding Bu-DIF-3 or CP-DIF-3 without changing the medium. After 24 h of incubation, the GFP/RFP ratio was measured as the reporter readout for autophagic flux, as described in Section 4.3.

#### 4.7.3. Measurement of MMP

MMP was evaluated using MitoBright LT Green and MT-1 MitoMP probes (Dojindo Laboratories, Kumamoto, Japan) as reported previously [53,54]. HeLa-WT cells were seeded at 2 × 10^5^ cells/dish in a 3.5 cm glass-bottom dish with 2 mL of medium. After 24 h, the cells were treated with 0.1% DMSO, 5 µM Bu-DIF-3, or 5 µM CP-DIF-3 for 24 h and then stained in accordance with the manufacturer’s instructions. Fluorescence images were captured using a 20× objective lens under a fluorescence microscope, and green (MitoBright LT Green) and red (MT-1 MitoMP) channels were quantified using BZ-810 Analyzer software (Keyence, Osaka, Japan). Relative MMP was calculated as the ratio of red MT-1 MitoMP fluorescence to green MitoBright LT Green fluorescence.

#### 4.7.4. Detection of Intracellular Labile Ferrous Iron (Fe^2+^) Signal

The intracellular labile Fe^2+^ signal was visualized using FerroOrange. HeLa cells were treated with vehicle, BPY (100 µM), Bu-DIF-3 (5 µM), or CP-DIF-3 (5 µM) for 24 h. For the BPY-treated group, BPY was removed before FerroOrange staining. The cells were washed and incubated with 1 µM FerroOrange in a serum-free medium at 37 °C for 30 min in the dark. Fluorescence was observed using an inverted fluorescence microscope with a SYTOX Orange filter set (Ex: 546 nm, Em: 565 nm), and the fluorescence intensity was quantified using the BZ-X800 Analyzer software (Keyence). For the BPY-treated group, BPY was removed before FerroOrange staining and was not included in the staining solution.

### 4.8. Cellular Metabolic Activity (MTT Assay)

HeLa cells were seeded at 5 × 10^3^ cells/well in 96-well plates and incubated for 24 h. The cells were treated with or without Bu-DIF-3 or CP-DIF-3 (5, 10, or 20 µM) for 24 h. For iron chelation assays, the cells were treated with 5 or 10 µM Bu-DIF-3 in the presence or absence of 25 µM DFO for 6 h, as described previously [55]. The 6 h window was selected to limit the influence of prolonged DFO exposure on the MTT readout. After the 24 h or 6 h treatment, the compound-containing medium was removed, and the cells were washed once with PBS. Each well then received 180 µL fresh drug-free medium and 20 µL MTT stock solution (5 mg/mL; final concentration, 0.5 mg/mL; Sigma-Aldrich, Merck KGaA, Darmstadt, Germany), followed by incubation for 4 h. The medium was then removed, and the formazan crystals were dissolved in 150 µL DMSO for 10 min with shaking before absorbance measurement. Absorbance was measured at 570 nm using a microplate reader (Thermo Fisher Scientific). Cellular metabolic activity was expressed as a percentage relative to the DMSO control. The MTT assays in Figure 6C,D were performed in three independent experiments, with five technical wells per condition in each experiment. Technical-well values were averaged within each experiment before calculating the mean ± SEM and performing statistical comparisons across the three independent experiments.

### 4.9. Statistical Analysis

All data were analyzed and plotted using Origin 2021 software. The results are presented as the mean ± SEM from at least three independent experiments. Statistical significance was determined using an unpaired two-tailed Student’s *t*-test for two-group comparisons. For comparisons involving three or more groups with one independent factor, one-way ANOVA followed by Tukey’s multiple-comparison test was used. For experiments involving two independent factors, two-way ANOVA followed by Tukey’s multiple-comparison test was used. The statistical test and exact number of independent experiments used for each analysis are specified in the corresponding figure legend. Statistical significance was set at *p* < 0.05. Significance levels are denoted as * *p* < 0.05, ** *p* < 0.01, and *** *p* < 0.001.

## 5. Conclusions

We identified Bu-DIF-3 and CP-DIF-3 as compounds that increased reporter-defined autophagic flux and observed accompanying AMPK activation, mTORC1 inhibition, and mitochondrial depolarization principally in HeLa cells, with selected autophagy and AMPK measurements performed in HAP1 cells. Bu-DIF-3 also elicited ROS accumulation, increased FerroOrange fluorescence consistent with an increase in the intracellular labile Fe^2+^ signal, and reduced cellular metabolic activity that was partially restored by DFO. The partial rescue supports the possible involvement of iron-sensitive processes but does not demonstrate that labile iron directly caused oxidative injury and does not establish ferroptosis. These findings indicate that structural modification of the DIF-3 scaffold influences the autophagy-reporter response and accompanying cellular stress response. Bu-DIF-3 may therefore serve as a useful chemical probe for studying the relationship among autophagy, mitochondrial dysfunction, and iron homeostasis and warrants further evaluation in additional cellular and in vivo models.

## Data Availability

The original data presented in the study are openly available in the NCBI Gene Expression Omnibus at (https://ncbiinsights.ncbi.nlm.nih.gov/tag/geo/, access date on 5 August 2026) or number GSE341456.

## Supplementary Materials

The following supporting information can be downloaded at: https://www.mdpi.com/article/10.3390/ijms27209380/s1.
