# Supplementary material for: Derivatives of Dictyostelium Differentiation-Inducing Factor-3 Promote Autophagic Flux, Mitochondrial Dysfunction and Iron-Associated Oxidative Stress
Source: Int J Mol Sci. 2026 Oct 21;27(20):9380. doi: 10.3390/ijms27209380 (PMC13705388; doi:10.3390/ijms27209380)
Supplement: Supplementary file 1 [file ijms-27-09380-s001.zip › Supplementary Tables and Figures.pdf]

Table S1. RNA-sequencing library, quality-control, alignment, and gene-assignment metrics for GSE341456.

HAP1-WT cells were treated with vehicle, Bu-DIF-3 (5 μM), or CP-DIF-3 (5 μM) for 24 h; each group comprised three independently prepared biological replicates.

Library ID	Treatment	Biological replicate	GEO sample	SRA run	Raw read pairs	Total bases (Gb)	Gene-assigned fragments	Gene assignment (%)
C1	Vehicle control	1	GSM9907078	SRR39848935	21,731,765	6.52	16,485,011	75.9
C2	Vehicle control	2	GSM9907079	SRR39848934	21,296,296	6.39	16,481,527	77.4
C3	Vehicle control	3	GSM9907080	SRR39848933	24,121,222	7.24	18,570,670	77.0
Bu1	Bu-DIF-3, 5 μM, 24 h	1	GSM9907081	SRR39848932	28,793,532	8.64	22,089,965	76.7
Bu2	Bu-DIF-3, 5 μM, 24 h	2	GSM9907082	SRR39848931	23,140,443	6.94	17,686,833	76.4
Bu3	Bu-DIF-3, 5 μM, 24 h	3	GSM9907083	SRR39848930	23,696,612	7.11	18,041,976	76.1
CP1	CP-DIF-3, 5 μM, 24 h	1	GSM9907084	SRR39848929	22,799,656	6.84	17,534,121	76.9
CP2	CP-DIF-3, 5 μM, 24 h	2	GSM9907085	SRR39848928	21,031,752	6.31	16,318,882	77.6
CP3	CP-DIF-3, 5 μM, 24 h	3	GSM9907086	SRR39848927	21,682,634	6.50	16,813,202	77.5

Notes. Libraries were sequenced as 150-bp paired-end reads on an Illumina NovaSeq 6000. Raw read-pair and base counts were obtained from SRA RunInfo (BioProject PRJNA1502797). Gene-assigned fragments were calculated as the sum of the deposited featureCounts gene-level counts for each library. Gene assignment (%) = gene-assigned fragments/raw read pairs × 100 and should not be interpreted as the overall HISAT2 mapping rate.

Additional per-library quality-control and alignment metrics were recovered from the original sequencing-provider project outputs (data_table.xls and align_pct.xls). The provider reports raw and clean reads as individual reads; accordingly, the SRA raw read-pair values in the table above equal one-half of the corresponding provider raw-read counts.

Per-library sequencing quality and alignment metrics

Library ID	Clean reads	Error rate (%)	Q20 (%)	Q30 (%)	GC (%)	Overall mapped (%)	Uniquely mapped (%)
C1	42,810,760	0.03	96.20	89.66	48.59	95.79	93.28
C2	41,980,048	0.03	96.93	91.33	49.16	96.37	93.81
C3	47,625,244	0.03	96.63	90.73	49.14	95.93	93.39
Bu1	56,860,822	0.03	96.55	90.54	48.92	95.67	93.03
Bu2	45,583,220	0.03	96.90	91.25	48.85	95.94	93.32
Bu3	46,705,000	0.03	96.64	90.70	48.93	95.76	93.12
CP1	44,903,346	0.03	96.61	90.63	49.32	95.86	93.27
CP2	41,430,058	0.03	96.77	90.93	48.85	96.45	93.88
CP3	42,762,702	0.03	96.63	90.73	48.79	96.29	93.74
**Mean**	**—**	**0.03**	**96.65**	**90.72**	**48.95**	**96.01**	**93.43**

Metric definitions. Q20, Q30, GC content, error rate, and clean-read counts were obtained from the project quality-control summary; overall and unique mapping rates were obtained from the HISAT2 alignment statistics. Means are arithmetic means across the nine libraries. Percentages are reported as supplied by the sequencing provider.

Table S2. Effects of DIF Derivatives on the AMPK–mTORC1 Pathway

Readout and condition	Bu-DIF-3	CP-DIF-3	Interpretation
Autophagic flux, 5 μM, 24 h	Larger decrease in GFP/RFP ratio	Smaller decrease in GFP/RFP ratio	Both enhance reporter-defined flux; Bu-DIF-3 is more potent under these conditions
AMPK and mTORC1 signaling	AMPK phosphorylation increased; mTORC1 readouts decreased	AMPK phosphorylation increased; mTORC1 readouts decreased	Parallel signaling changes; AMPK dependence was not tested
Mitochondrial membrane potential, 5 μM, 24 h	Significant decrease; greater effect	Significant decrease; smaller effect	Both induce mitochondrial depolarization
CellROX signal, 5 μM, 24 h	Significant increase	Non-significant tendency	Redox response is more pronounced with Bu-DIF-3
FerroOrange signal, 5 μM, 24 h	Significant increase	No significant increase	Labile Fe^{2+} response detected only with Bu-DIF-3 at the tested concentration
MTT-derived metabolic activity, 5–20 μM	Concentration-dependent decrease	Concentration-dependent decrease	MTT does not distinguish cytostasis, altered metabolism, and cell loss
DFO co-treatment	Partial attenuation of reduced MTT signal	Not evaluated	Supports an iron-sensitive component for Bu-DIF-3; rescue is incomplete

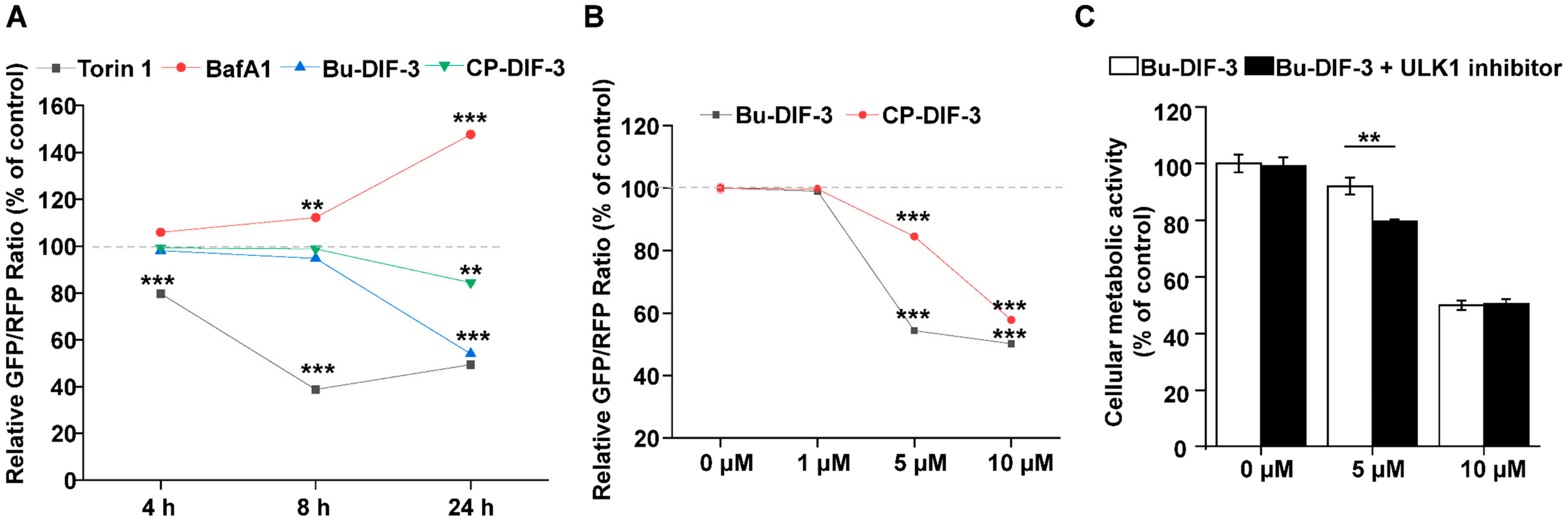

Figure S1. Time- and concentration-dependent autophagy-reporter responses and the effect of MRT68921 co-treatment on the Bu-DIF-3-associated MTT response. (A) HeLa-GFP-LC3-RFP-LC3ΔG cells were treated with Torin1 (1 μM), BafA1 (200 nM), Bu-DIF-3 (5 μM), or CP-DIF-3 (5 μM) for 4, 8, or 24 h. (B) Reporter cells were treated with Bu-DIF-3 or CP-DIF-3 at 0, 1, 5, or 10 μM for 24 h. GFP/RFP ratios were normalized to the matched vehicle control. (C) HeLa-WT cells were treated with Bu-DIF-3 (0, 5, or 10 μM) with or without MRT68921 (1 μM) for 24 h, and the MTT signal was normalized to the matched vehicle control. Data are presented as mean ± SEM from three experiments ($n = 3$). Statistical significance was determined using a two-tailed Student's t-test; $**p < 0.01$ and $***p < 0.001$.

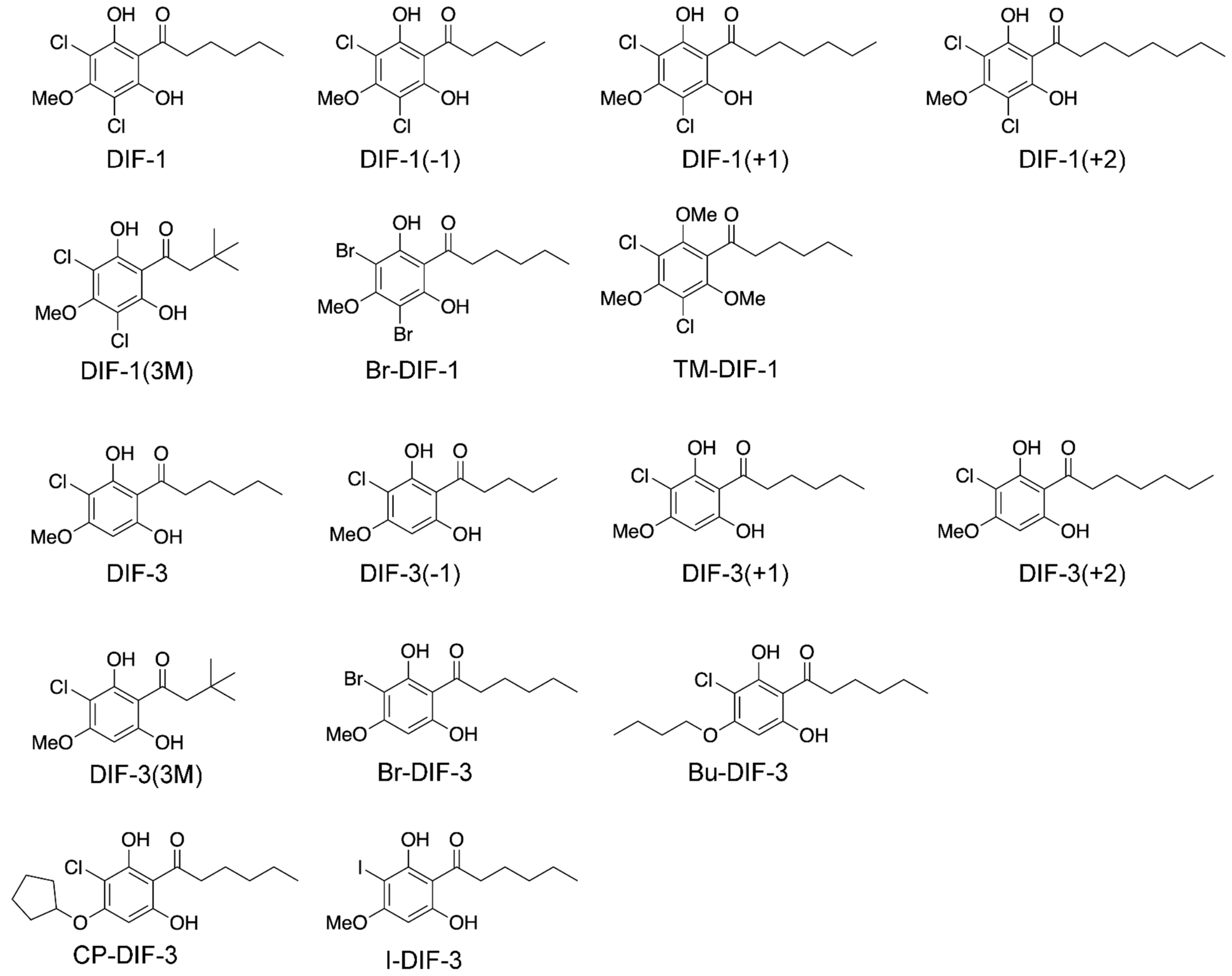

Figure S2. Chemical structures of DIF compounds used in this study.

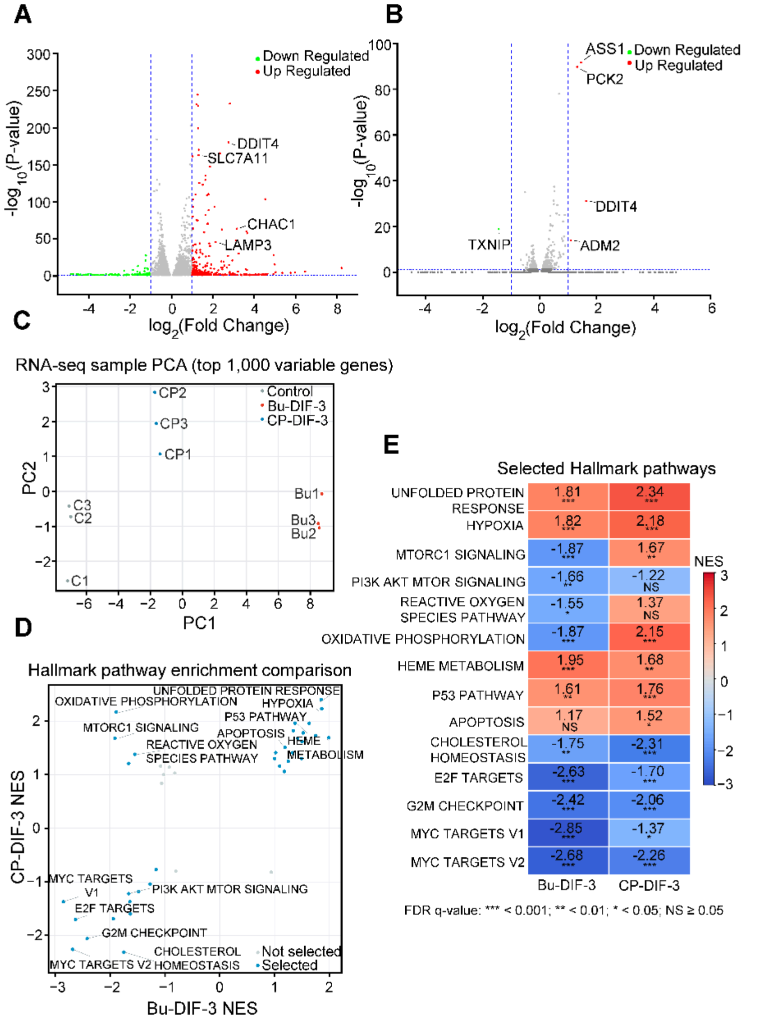

Figure S3. Differential expression and threshold-independent RNA-sequencing analyses of Bu-DIF-3- and CP-DIF-3-treated cells. (A, B) Volcano plots comparing Bu-DIF-3 and CP-DIF-3, respectively, with vehicle control. Differentially expressed genes were defined as $|\log_2$ fold change$| > 1$ and padj < 0.05; the vertical dashed lines mark $\log_2$ fold changes of -1 and $+1$, and the horizontal dashed line marks padj $= 0.05$. Upregulated and downregulated genes are shown in red and green, respectively. (C) Principal-component analysis of the top 1,000 variable genes across three vehicle-control, three Bu-DIF-3, and three CP-DIF-3 biological replicates. (D) Comparison of normalized enrichment scores (NES) from pre-ranked Hallmark gene set enrichment analysis (GSEA). (E) Heatmap of selected Hallmark NES values. Panels C–E were generated without applying a differential-expression or fold-change cutoff; GSEA used complete gene lists ranked by the DESeq2 Wald statistic. Benjamini–Hochberg FDR q-values are shown in panel E; $***q < 0.001$, $**q < 0.01$, $*q < 0.05$, and NS $q \geq 0.05$.

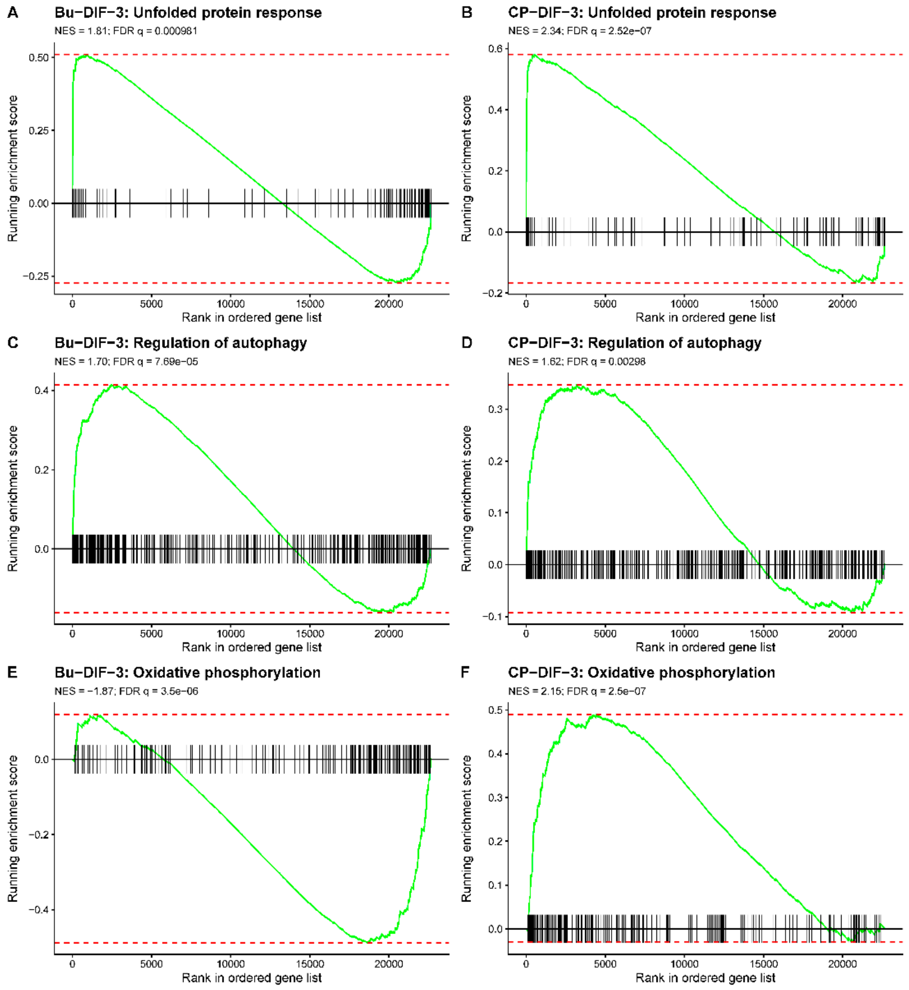

Figure S4. Threshold-independent gene set enrichment analyses for Bu-DIF-3 and CP-DIF-3. (A, B) Hallmark unfolded-protein-response enrichment for Bu-DIF-3 and CP-DIF-3, respectively. (C, D) GO Biological Process regulation-of-autophagy enrichment for Bu-DIF-3 and CP-DIF-3, respectively. (E, F) Hallmark oxidative-phosphorylation enrichment for Bu-DIF-3 and CP-DIF-3, respectively. Each analysis used the complete gene list ranked by the DESeq2 Wald statistic without applying a differential-expression or fold-change cutoff. NES and Benjamini–Hochberg FDR *q*-values are shown in each panel. Complete Hallmark and GO Biological Process results and analysis parameters are provided in Supplementary Data S1.
